# Zoonotic Cryptosporidiosis from Petting Farms, England and Wales, 1992–2009

**DOI:** 10.3201/eid1701.100902

**Published:** 2011-01

**Authors:** Fraser J. Gormley, Christine L. Little, Rachel M. Chalmers, Nalini Rawal, Goutam K. Adak

**Affiliations:** Author affiliations: Health Protection Agency, London, UK (F.J. Gormley, C.L. Little, N. Rawal, G.K. Adak);; Public Health Wales, Swansea, UK (R.M. Chalmers)

**Keywords:** Cryptosporidium, enteric infections, petting farms, diarrhea, risk, E. coli, parasites, zoonoses, England, Wales, letter

**To the Editor:** Visits to petting farms in England and Wales recently have increased in popularity. Petting farms are commercial operations at which visitors, mainly families and organized groups, are encouraged to have hands-on contact with animals. The ≈1,000 petting farms in the United Kingdom collectively receive >2 million visitors per year, with peak visitor times during school and public holidays. Commercial farms also may host farm visits on single days for group and school visits. The farm attraction business is a substantial part of the rural economy, generating >£12 million annually (*1*).

During 1992–2009, a total of 55 outbreaks of infectious intestinal disease associated with petting farms in England and Wales was reported to the Health Protection Agency. Verocytotoxin-producing *Escherichia coli* O157 (VTEC O157) caused 30 (55%) of these outbreaks (244 persons were affected [range 2–93, mean 8 persons] and 84 were hospitalized); *Salmonella enterica* serovar Typhimurium definitive phage type 104 caused 2 (3%) of the outbreaks. A total of 23 (42%) petting farm outbreaks were caused by *Cryptosporidium* spp. (1,078 persons were affected [range 2–541, mean 45 persons] and 29 were hospitalized). We report on these cryptosporidiosis outbreaks as a reminder of the risk to petting farm visitors.

Contributory factors reported in the cryptosporidiosis outbreaks included direct contact with preweaned lambs, calves, kids, or animal feces (e.g., diarrhea in lambs, a recognized risk factor for cryptosporidiosis; 11/23 [48%]) and inadequate hand washing facilities (7/23 [30%]). Of outbreaks in which hand washing facilities were inadequate, thumb sucking by children was also noted in 1; in another, alcohol-based hand gels and sanitizers, which are ineffective against *Cryptosporidium* **spp.**, were used.

***Cryptosporidium* spp.** are coccidian parasites **that** infect a wide range of farm livestock, including cattle, sheep, goats, pigs, horses, and deer, but are mainly a veterinary problem in neonatal ruminants. *C. parvum*, for example, is a common agent in the etiology of the neonatal diarrhea syndrome of calves, lambs, and goat kids. Widespread asymptomatic carriage of this parasite exists in livestock in the United Kingdom (*2*). In humans, cryptosporidiosis occurs most commonly in children <5 years of age, can be life threatening in immunocompromised persons, and is caused predominantly by *C. hominis* and *C. parvum* parasites. Fecal–oral transmission can occur directly from animal to person and from person to person or indirectly through contaminated food or water (*2*).

Typing of *Cryptosporidium* spp. has been undertaken by the UK *Cryptosporidium* Reference Unit since 1999. *C. parvum* was identified from human feces in 12 (75%) of the 16 petting farm outbreaks since 1999 (feces were not submitted for typing in 4). Additionally, *Cryptosporidium* spp. oocysts were detected and confirmed as *C. parvum* from suspected sources (lambs, calves) in 4 (33%) of these 12 outbreaks and linked by GP60 subtype to human cases in 3 outbreaks. Zoonotic risk factors in case–control studies of sporadic cryptosporidiosis cases in England and Wales also have identified an association between *C. parvum* infection and touching farm animals or visiting a farm (*3*).

In petting farm outbreaks, *Cryptosporidium* spp. displayed a seasonal pattern, as did VTEC O157. Cryptosporidiosis outbreaks occurred more often in springtime (18 vs. 5; p = 0.0001) than did VTEC O157 outbreaks, which occurred more frequently during the summer (25 vs. 5; p<0.00001), especially in August (Figure). During spring 2010, two additional *C. parvum* outbreaks associated with contact with lambs at petting farms were reported in England. Control measures included restricting bottle feeding of lambs and enhancing the supervision of hand washing. The associations with outbreaks of cryptosporidiosis in spring and contact with young farm animals also has been reported in Scotland (*4*).

**Figure:**
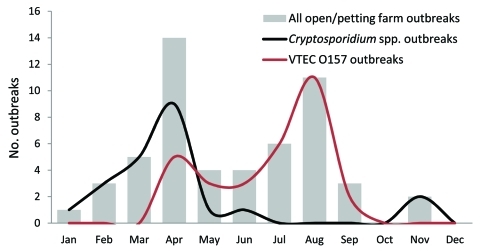
Outbreaks of cryptosporidiosis and verocytotoxin-producing *Escherichia coli* O157 linked to petting farms, England and Wales, 1992–2009.

Despite the 2 separate seasonal peaks of infection, care should be exercised throughout the year. The importance of careful attention to hygiene and supervision of children visiting farms and the need for appropriate facilities, such as those for hand washing, are covered in the UK Health and Safety Executive standards; operators of petting farms are expected to meet these standards (*5*). These guidelines also apply to commercial farms hosting open days. A good practice reminder on managing the risks from VTEC O157 in a petting farm context was published by the Health Protection Agency, Health and Safety Executive, and the Local Government Regulation (*6*). Guidance on the control of VTEC O157 infections for farms open to public access applies equally to most gastrointestinal pathogens, including *Cryptosporidium* spp. The need for a sound approach to managing hygiene control measures at petting farms cannot be overemphasized.
